# Cognitive mechanisms for inferring the meaning of novel signals during symbolisation

**DOI:** 10.1371/journal.pone.0189540

**Published:** 2018-01-16

**Authors:** Justin Sulik

**Affiliations:** 1 Max Planck Institute for Psycholinguistics, Nijmegen, Netherlands; 2 Department of Psychology, Royal Holloway, University of London, London, United Kingdom; Fordham University, UNITED STATES

## Abstract

As participants repeatedly interact using graphical signals (as in a game of Pictionary), the signals gradually shift from being iconic (or motivated) to being symbolic (or arbitrary). The aim here is to test experimentally whether this change in the form of the signal implies a concomitant shift in the inferential mechanisms needed to understand it. The results show that, during early, iconic stages, there is more reliance on creative inferential processes associated with insight problem solving, and that the recruitment of these cognitive mechanisms decreases over time. The variation in inferential mechanism is not predicted by the sign’s visual complexity or iconicity, but by its familiarity, and by the complexity of the relevant mental representations. The discussion explores implications for pragmatics, language evolution, and iconicity research.

## Introduction

**Fig 1:**
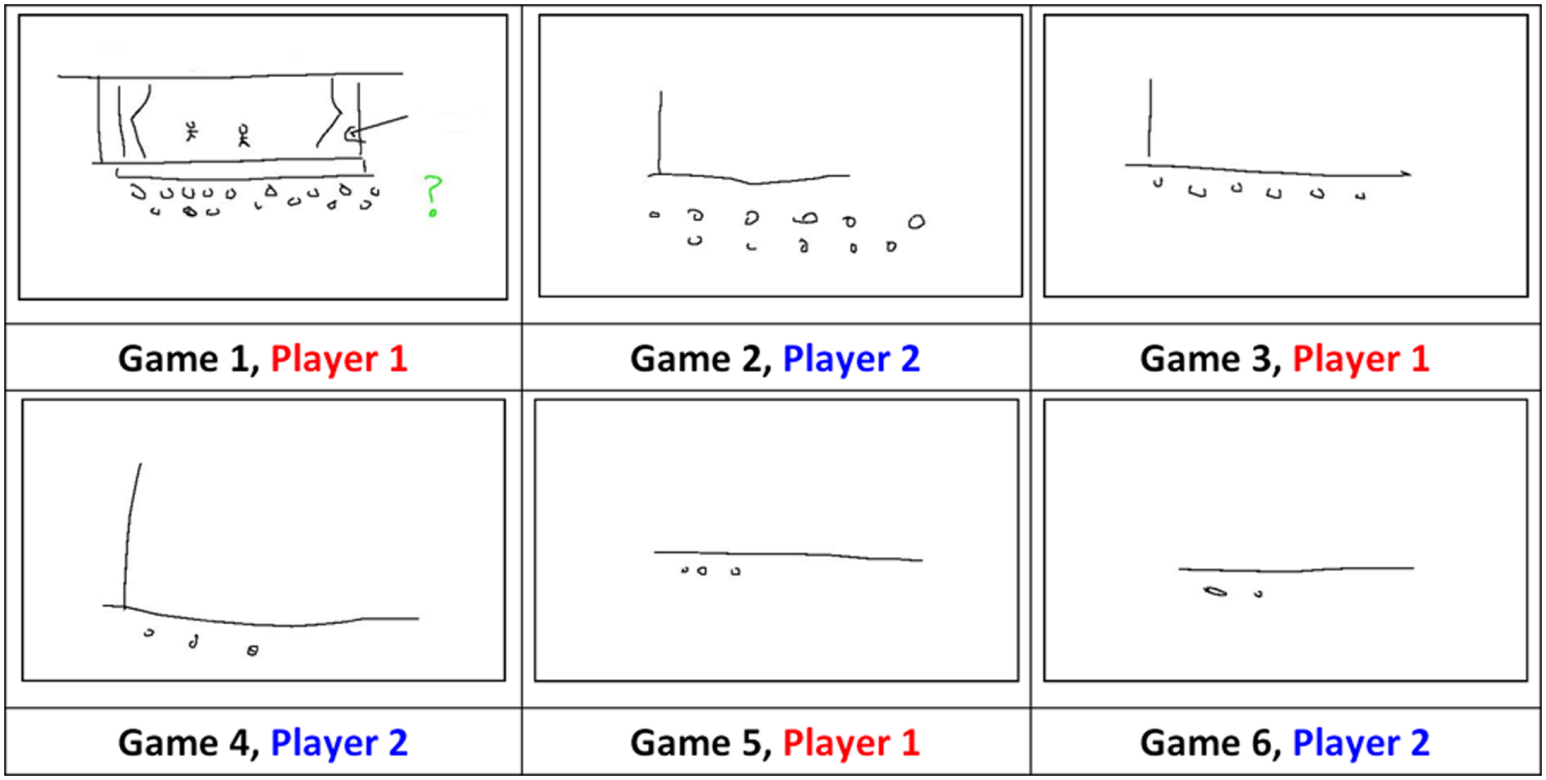
Over several rounds (‘games’) of repeated interaction in a novel signalling task, an iconic sign for ‘theatre’ becomes simplified or more abstract, resulting in a conventional or symbolic sign (drawn from a corpus collected by [2], published in PLoS ONE by [4]; use permitted under the Creative Commons Attribution License CC BY 4.0).

Humans are readily able to generate hypotheses about the meanings of novel signals in communicative games such as charades or Pictionary (hereafter, ‘novel signalling tasks’), even when the perceptual form of these signals changes over time. For instance, Garrod, Fay and colleagues [1, 2] investigate repeated Pictionary-like games, where participants draw graphical representations of cues which a partner has to guess, and where this process is repeated over several rounds with the same items. They show that, if the participants are able to interact while playing, the initially iconic signs become less iconic or more conventionalised, resulting in symbolic signs (iconic signs share perceptual properties with their referents, whereas symbolic signs are arbitrary, lacking such a link, or are those for which convention or habit play a role in their interpretation [3]). This shift from iconic to symbolic signs is called **symbolisation**, and the process is exemplified in Fig 1.

Research in symbolisation typically focuses on how the perceptual form of the signal varies over repeated rounds, or how interpersonal interaction affects this. A question not yet tackled by the literature is what cognitive mechanisms are recruited during symbolisation. The central questions investigated here, then, are (1) whether the cognitive mechanisms underlying inference about meaning at earlier iconic stages of symbolisation are different from those during later, less iconic stages; and if so, (2) what the relevant cognitive mechanisms are; and (3) what features of the task predict such a difference.

Answers to these questions would help fill a gap in our understanding of the evolution of language in our species. Prior to the evolution of human symbolic language, there may have been an iconic protolanguage stage [5–9]. In this view, our ancestors first developed iconic signs, and the process of symbolisation eventually lead to these becoming conventional or arbitrary. If symbolisation is part of the explanation for how our species evolved to communicate linguistically, and since we are unsure just what cognitive processes are involved at various stages of symbolisation, then a serious gap remains in our understanding of one of the defining traits of our species

## Background

### Symbolisation

Garrod et al. [1] explore the conditions under which symbolisation happens. The basic set-up of their experiment 1 was as follows (‘basic’ in that this is the point of departure for the experiment described below). Participants played six rounds of a novel signalling task. In each round, one participant (the director or signaller) was given a list of target items and, one-by-one, produced drawings to help the other participant (the matcher or receiver) guess each item. Items included easily confusable places, people, TV or film genres, objects and abstract words. The use of speech or letters was prohibited. Receivers were provided with the full list of items to aid guessing. After all items in a round were guessed, the participants swapped roles and repeated the game with the same items.

Garrod et al. [1] found that when feedback was allowed, the signs became simpler and less iconic (Fig 1). They frame this change in informational terms, arguing that icons carry information in their graphical structure. As the signs become less iconic, this informational burden shifts from the signal to the receiver. During earlier stages in Fig 1, the signal is interpreted because of what the receiver *sees*, whereas in later stages, it is interpreted because of what the receiver *knows* about previous interactions, and at this stage the signal itself is less informative. Symbolisation, in this view, is characterised as a gradual shift in informational burden from what the receiver perceives to what they know.

### Icons

**Fig 2:**
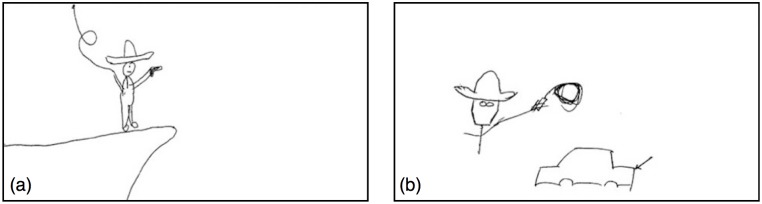
Pictures produced independently by different signallers during experiment 1, below, representing the cue ‘Harrison Ford’.

Icons involve resemblance, but not all icons are interestingly alike from a cognitive point of view. Consider Fig 2, two novel signals drawn independently by two participants in study 1 below to represent the cue ‘Harrison Ford’. Imagine comparing these to a realistic painting of Harrison Ford. A realistic painting would straightforwardly look like Harrison Ford, and we would recognise him because of how it looks, because of the information it carries in its graphical structure [1].

On the other hand, although the novel signals in Fig 2 are iconic, they don’t straightforwardly resemble Harrison Ford. Unlike the painting, one cannot simply tell by looking that it is him. Of all the possible facts about him (he’s American, he’s an actor, he’s male and caucasian, he’s married to Calista Flockhart, etc.) both signallers have independently settled on the fact that he played Indiana Jones, and of all the possible facts about Indiana Jones (he’s an archaeologist, he’s a professor, he’s afraid of snakes, etc.) the signallers have settled on the fact that he carries a whip and has a distinctive hat. Additionally, the signaller in Fig 2b has construed Harrison Ford as sharing a name with a make of car. From the signallers’ perspectives, these are salient aspects of Harrison Ford. These salient aspects of the signallers’ world knowledge that have been foregrounded in the signal are called its **ground** [10, 11].

The ground here is not directly communicated to the receiver or straightforwardly perceived by them. Rather than simply *recognising* Harrison Ford, the receiver has to *infer* the signaller’s communicative intentions. The signal is just the input to an inferential process whereby the receiver must use their world knowledge to generate the hypothesis that the cue was Harrison Ford [12, 13]. The hat in Fig 2a may look like a sombrero, but trying to interpret it this way is a dead-end. Rather, it is a poor representation of a fedora, and understanding this is part-and-parcel of inferring the meaning of the whole. Thus, the receiver cannot take the relevance of any part of the signal for granted, but rather must infer the relevance of all these elements, so call this a **relevance-deciding problem**.

This suggests an alternative way to characterise symbolisation. Garrod et al. [1] described it as involving a shift from what the receiver perceives to what they know. However, in another (possibly complementary) sense, the receiver’s knowledge is crucial throughout. In earlier iconic stages, the receiver has to infer the relevance of the ground, based in part on what they know about the world. When those elements reappear later on, the receiver simply has to recognise them, based on what they know about the history of use of those elements. For instance, in earlier rounds in Fig 1, the receiver has to infer that the dots at the bottom represent the audience. In later rounds, they simply recognise the signal as being similar to ones seen earlier.

This has implications for the main research question: are different cognitive mechanisms needed throughout symbolisation? The most parsimonious hypothesis is that we rely on the same cognitive mechanisms throughout. Perhaps it is sufficient to describe symbolisation as requiring inductive inference and leave the matter there. The alternative hypothesis suggested here is that different cognitive mechanisms are needed at different stages. Specifically, in earlier stages, a heavier burden will be placed on whatever cognitive mechanisms we bring to bear on relevance-deciding problems and in later stages, less of a burden.

The question, in a nutshell, is what happens, cognitively, when people *don’t* recognise a signal (i.e. when it is new, before it conventionalises)? However, before the matter can be put to experimental test, two issues need to be resolved: firstly, what makes something a relevance-deciding problem? Secondly, what are the cognitive mechanisms we bring to bear on relevance-deciding problems, and how would we tell that they are in fact being brought to bear on a particular problem?

### Relevance-deciding problems

The broad hypothesis under consideration is that different cognitive mechanisms are needed at various stages of symbolisation. A more specific hypothesis is that relevance-deciding mechanisms are important earlier on, and less so later. We thus need a way to decide empirically if a particular signal or item presents a relevance-deciding problem. If the broad hypothesis turned out to be true, then a change in the degree of iconicity would be a parsimonious explanation for a change in processing, since we already know that signals become less iconic during symbolisation [1, 2]. To support the specific alternative hypothesis, we must show that something other than iconicity predicts the recruitment of particular mechanisms of interpretation.

When a receiver first sees something like one of the circles in Fig 1, they have to infer its relevance. Later on, they simply have to recognise it as an instance of a problem they’ve already solved. The signal’s *novelty* (or conversely, its level of *familiarity*) should thus be a factor in determining the cognitive mechanisms needed for its interpretation. Although novelty and iconicity may be correlated (during symbolisation, iconicity decreases as the participants become more familiar with the signals), they are nonetheless distinguishable. For instance, it is plausible that a signal in round *t* might be accurately reproduced in round *t* + 1. In that case, its familiarity increases while its iconicity remains constant. Alternatively, if a signal in round *t* proves ineffective (e.g., it takes a long time to guess), the signaller might change tack in round *t* + 1 and try a different construal (say, represent Harrison Ford as Han Solo instead of Indiana Jones). In that case, the iconicity of the new signal in round *t* + 1 may be high or not, whereas its novelty will definitionally be high, and the receiver will have to infer its relevance all over again. Thus, in addition to measuring a signal’s iconicity, it is necessary to measure whether it is novel or familiar, relative to signals drawn in previous rounds of the game.

However, novelty alone does not mean that something is a relevance-deciding problem. A second factor follows from the distinction between relevance-based meaning and code-like meaning [12]. In a code, an element in one plane (e.g., meaning space) corresponds with an element in another plane (e.g., signal space) in a rule-like, predictable way [14]. In Morse code, for instance, there is a rule-like relationship between particular letters and particular sequences of dots and dashes. Sperber and Wilson [12] acknowledge that a code model may account for some aspects of language, but argue that many other aspects (such as pragmatics) require something more: open-ended inferential processes of the sort described in the previous section.

If Sperber and Wilson [12] are right, then we should be able to derive a measure of whether something presents a relevance-deciding problem by evaluating the degree to which the signal is predictable given the target item or concept. At the extreme code-like end of this continuum, letter S is predictably rendered as three dots in Morse code. Similarly, for some target items in a novel signalling task, the range of construals might be relatively constrained, in which case the signal should be predictable from the target concept, and independently drawn pictures should thus be similar to each other. Moving away from a code-like situation, the signallers who drew Fig 2 were able to choose between several grounds. One construed Ford as a make of car, and others could plausibly construe him as Han Solo, for instance. At the extreme relevance-deciding end of the continuum, the range of construals might be vast, such that there is little or no overlap in the signals’ grounds. The less predictable the ground from the concept, the less similarity between pictures drawn independently for that concept, and the more of a relevance-deciding problem posed by the item.

To illustrate, both drawings in Fig 2 represent Harrison Ford as a man with a hat and a whip. This is thus a somewhat predictably salient feature of Harrison Ford. If these figures are tagged for informative or meaningful elements, Fig 2a can plausibly be tagged as {MAN, HAT, CLIFF, GUN, WHIP} and Fig 2b as {MAN, HAT, WHIP, CAR} (for details of the tagging schema see Methodology, and Open Science Framework material). If each pair of signals drawn for a given meaning is represented by sets of tags X and Y, then the similarity between the signals’ grounds is just the proportion of shared elements (Jaccard Index, JI: the size of the intersection of the two sets divided by the size of the union). The difference between the grounds (Jaccard Distance, JD) is just one minus the Jaccard Index:

$$\begin{array}{c} \text{JD}=1-\text{JI}=1-\frac{\left|X\cap Y\right|}{\left|X\cup Y\right|} \end{array}$$ (1)

Thus, the JD between the two signals in Fig 2 is $1-\frac{3}{6}=0.5$ since 3 tags {MAN, HAT, WHIP} are shared out of the set of 6 tags {MAN, HAT, WHIP, CLIFF, GUN, CAR}. The JDs between all pairs of signals drawn for a target item can be averaged to provide a mean Jaccard Distance for the item (JD_*μ*_). This represents how much of a relevance-deciding problem that item poses. In a Morse-code-like situation, JD_*μ*_ = 0 since the signals are always precisely the same, whereas if the signals do not overlap at all, JD_*μ*_ = 1. To be clear: the intention is not to show that some graphical signals are literally codes. Rather, it is to show that, for some items, the patterns of salience that constitute the ground are relatively more predictable, and others less so. In that case, some cue items pose more of a relevance-deciding problem than others. We can thus test whether JD_*μ*_ plays a role in symbolisation that does not reduce to an effect of iconicity.

### Relevance-deciding cognitive mechanisms

The above factors identify a relevance-deciding *problem*, but we still need some way to identify relevance-deciding *cognitive mechanisms*, and to show that they are in fact being brought to bear on the interpretation of a signal during a novel signalling task.

A way of doing just that has been identified in the literature on insight problem solving. An insight problem is one where finding the relevant representation of the problem is hard [15]. Insight problems are thus hard relevance-deciding problems. Since there are well studied behavioural and neurological hallmarks of the recruitment of insight problem solving mechanisms, these can serve as diagnostics here: the more relevance-deciding a novel signalling problem is, the more likely participants should be to display such hallmarks.

In non-insight (i.e., analytic) problem solving, such as doing long division by a set of learnt rules or reasoning through a deductive syllogism, one begins with an appropriate representation of the problem, and is able to mechanically follow an algorithm that leads step-by-step to the solution [16, 17]. By contrast, in insight problem solving, it is not obvious what information is relevant to the solution, and the most obvious reading of the problem is typically misleading (often resulting in an impasse [17, 18]). The main difficulty is finding a relevant representation or construal in the first place, sometimes out of an open-ended set of possible problem dimensions [15].

An example is this: ‘A man in a small town married 20 different women of the same town. All are still living and he never divorced. Polygamy is unlawful but he has broken no law. How can this be?’ [19]. Solution requires one to restructure one’s representation of the problem [20], i.e., to suppress a predictable but misleading interpretation and search for an alternative construal (here, an alternative construal of the term ‘married’, since the solution is that the man officiated at the weddings). The existence of multiple construals here is precisely what the previous section identified as a feature of relevance-deciding problems. All that makes *this* an insight problem is that the relevant construal (as in ‘the clergyman married the bride and groom’) is much less probable given the word ‘married’ than the obvious but misleading construal (as in ‘the groom married the bride’), so the latter is difficult to override.

A hallmark of insight problem solving is that the solution is often accompanied by a sudden, distinctive ‘Aha!’ experience [17, 21], like a lightbulb metaphorically turning on in one’s head [22]. Crucially, evidence from neuroimaging [23] and divided-visual-field priming [24] shows that subjective reporting of an ‘Aha!’ experience correlates with measurable differences in cognitive processing. Subjective reporting of such an experience thus serves as a diagnostic for the recruitment of insight problem solving mechanisms: the more of a burden is placed on relevance-deciding mechanisms, the more likely participants are to report an ‘Aha!’ experience.

There is ample evidence distinguishing insight processing from more analytic processing, and linking it to key problem features outline above (contrasting predictable, familiar problems and less predictable, novel problems). The brain networks recruited during insight problem solving (centred on the right hemisphere/RH temporal lobe, [25]) are able to activate a relatively broad range of less predictable associates, whereas the corresponding left hemisphere (LH) regions are more likely to be successful when a smaller range of more predictable associates suffice for solution. These RH regions are more active when processing novel metaphors (which involve making creative, relevance-deciding inferences) whereas LH regions are more active for conventional (i.e., predictable and familiar) metaphors and literal phrases [26]. As novel metaphors become more familiar, there is a corresponding shift in processing [27].

Further, the same RH brain networks are active during novel signalling tasks [28, 29], and are implicated in various forms of pragmatic inference, such as understanding speaker intention [30], generating coherence-creating inferences [31, 32], creating representations of discourse-level meaning [33, 34], and inferring meanings that are less predictable from context [35, 36] or when the context is less constrained [37, 38].

In sum, insight problem solving involves a distinctive manner of processing. The cognitive mechanisms recruited by insight have been shown to be active in relevance-deciding inference, and are recruited in cases of novelty and unpredictability. Subjective reporting of an ‘Aha!’ experience is diagnostic of recruitment of these mechanisms. The claim is *not* that an ‘Aha!’ experience always happens or only happens for hard relevance-deciding problems, or that all relevance-deciding problems involve insight. Rather, insight problems lie on the opposite of the continuum from analytic, algorithmic, code-like problems [39], and the more of a burden that is placed on relevance-deciding mechanisms in a novel signalling task, the more likely it is that participants report an ‘Aha!’ experience.

### Summary and predictions

When the appearance of a signal changes over the time course of symbolisation, it is currently unclear whether there is a corresponding change in the cognitive mechanisms needed for interpretation. The most parsimonious hypothesis is that there isn’t (perhaps it’s all just inductive inference). *If* there is a change in cognition, the most parsimonious hypothesis is that it should be predicted by the signal’s iconicity or visual complexity, since we already know they decrease over time. However, the alternative hypothesis argued for here is that it should depend instead on the extent to which the signal presents a relevance-deciding problem. Such problems are characterised by a high degree of novelty, and by less predictable patterns of salience in the ground. Subjective reporting of an ‘Aha!’ experience is diagnostic of recruitment of relevance-deciding cognitive mechanisms.

In Experiment 1, a novel signalling task is repeated over several rounds. After guessing the meaning of each signal, the receiver reports the extent to which they had an ‘Aha!’ experience. The prediction is that participants should be more likely to report an ‘Aha!’ flash of insight in earlier stages of the symbolisation process, and less likely to do so in later stages, indicating that insight (and thus relevance-deciding mechanisms) play an important role in earlier stages and less so in later stages. Experiment 2 involves the collection of measures of iconicity, of novelty, and of how predictable the ground is (JD_*μ*_), to test what features of a particular novel signalling problem predict the kind of cognition needed to solve it.

## Experiment 1: Insight in graphical communication

### Overview

Pairs of participants took part in a graphical novel signalling task, and reported the extent to which they experience an insight ‘Aha!’ moment after each guess. The aim was to test whether the change in a signal during symbolisation corresponds with a change in the cognitive mechanisms needed to interpret it. The prediction is that the insight ratings will be higher at the beginning than at the end of the symbolisation process, indicating a shift in the cognitive processing.

### Methodology

#### Participants

20 University of Edinburgh students were recruited via the university’s job website (17 female; mean age = 20.08, SD = 1.86). All participants gave written, informed consent and were paid £6 for their participation. One pair was excluded from analysis because one participant repeatedly wrote English words on her drawings, despite instructions to the contrary. Ethics approval was obtained under the University of Edinburgh Linguistics and English Language Ethics committee procedures.

#### Materials

The cues were based on Fay et al. [2], who used 20 cue items divided into 5 categories (places, people, entertainment, objects, abstract) and some categories included a distractor. Because distractors might be more salient, and because salience is a confound, the distractors were removed and the categories were expanded to include 5 items each (Table 1). The abstract category in Fay et al. comprised both nouns and adjectives, but it was limited to nouns here, for the sake of uniformity.

**Table 1:** Items grouped by category.

Places	Actors	Entertainment	Objects	Abstract
theatre	Robert De Niro	drama	television	noise
art gallery	Arnold Schwarzenegger	soap opera	computer monitor	depression
museum	Clint Eastwood	cartoon	microwave	poverty
parliament	Chuck Norris	horror	window	nausea
university	Harrison Ford	sci-fi	iPad	violence

Each participant in a pair had their own response booklet. Each page in the booklet corresponded to one round, and the page for that round indicated which role (signaller or receiver) each participant was to take. The signaller’s page listed items to be signalled during that round. The receiver’s page had spaces for them to record their guess for each picture, in addition to a 7-point scale for them to provide an insight rating (by circling the relevant number on the scale), indicating the extent to which they had an ‘Aha!’ experience while guessing. The scale increased from left to right, such that circling 7 indicated a strong feeling of insight.

A shortlist of 10 items was randomly generated for each pair from the 25 items in Table 1. The 10-item shortlist constituted the list of cues for the signaller in round 1, and the order of the shortlist was randomised again before each subsequent round. Each game thus only involved a random subset of items, so items were not presented a consistent number of times across participant pairs. For instance, due to the random allocation, only two pairs saw item ‘art gallery’, while six saw item ‘Arnold Schwarzenegger’.

It is plausible that participants might find the game increasingly repetitive, and thus increasingly unlikely to provoke an insight response. Therefore, it is worth being able to check whether any change in insight ratings represents a response to individual items, or to the game as a whole (i.e., a fatigue effect). Consequently, every second round, one item from the shortlist was replaced by another from Table 1. This occurred every second round so that both participants in a pair would have the same number of turns with each item. Since new items appeared throughout the game, the analysis will be able to check whether any change in insight ratings reflects increasing familiarity with the game as a whole, or with individual items. Further, the regular inclusion of novel items means that participants cannot be certain just which items will appear in a given round.

#### Procedure

Pairs of participants played eight rounds of a Pictionary-like game, alternating role after each round, thus having four turns at each role. The progress of the game can thus be measured in terms of eight rounds or four turns. A coin toss decided which participant was signaller first.

The participants read instructions describing insight in terms of an ‘Aha!’ experience, like a light bulb suddenly flashing on in the head [22]. To give them the chance to experience an ‘Aha!’ moment before the experiment started, they attempted four typical insight tasks (Compound Remote Associate problems from [40]), and were told that the ‘Aha!’ experience is common when solving such problems. Participants were instructed not to include any English words, numbers, letters or other conventional signals in their drawings. They were able to ask questions if anything was not clear. When both participants indicated they were ready to proceed, they were given the list of 25 possible answers and were allowed a minute to familiarise themselves with the items.

The signaller then read the first cue on the 10-item shortlist for round 1 in their booklet, and began drawing a picture to help the receiver guess the cue. The signaller was required to continue drawing until the receiver said ‘stop’, indicating that they had guessed. No information passed between signaller and receiver except the signal itself, and the receiver’s saying ‘stop’. Thus, the signaller did not know what the receiver had guessed, and the receiver did not know if their guess was correct. Throughout the whole process, the signaller and receiver both had access to the table of 25 items.

The receiver recorded their guess on their response sheet and also recorded an insight rating indicating the extent to which they had experienced an ‘Aha!’ moment. The signaller then proceeded to the next item on the list. This process repeated until all 10 items for that round had been guessed. At that point, both participants turned to a new page, which indicated that they should swap roles. The procedure for each round was identical, apart from the role played by each participant.

### Analysis

The analysis reports models fitted with the lmer or glmer functions (for linear and generalised linear mixed-effects models, respectively) from the lme4 package [41] in R [42]. Since the lmer function does not provide p-values [43], two approaches to significance are taken here: (1) p-values are calculated using the lmerTest package [44], and (2) bootstrapped 95% confidence intervals (CIs) for the estimated fixed effects are derived using the bootMer function from the lme4 package.

The random effects structure of the models reported below is the maximal structure justified by experiment design [45], *except* in cases of non-convergence or overparameterisation [43]. Thus, unless otherwise stated, the default structure includes both random slopes and intercepts for item and participant.

### Results

#### Symbolisation

**Fig 3:**
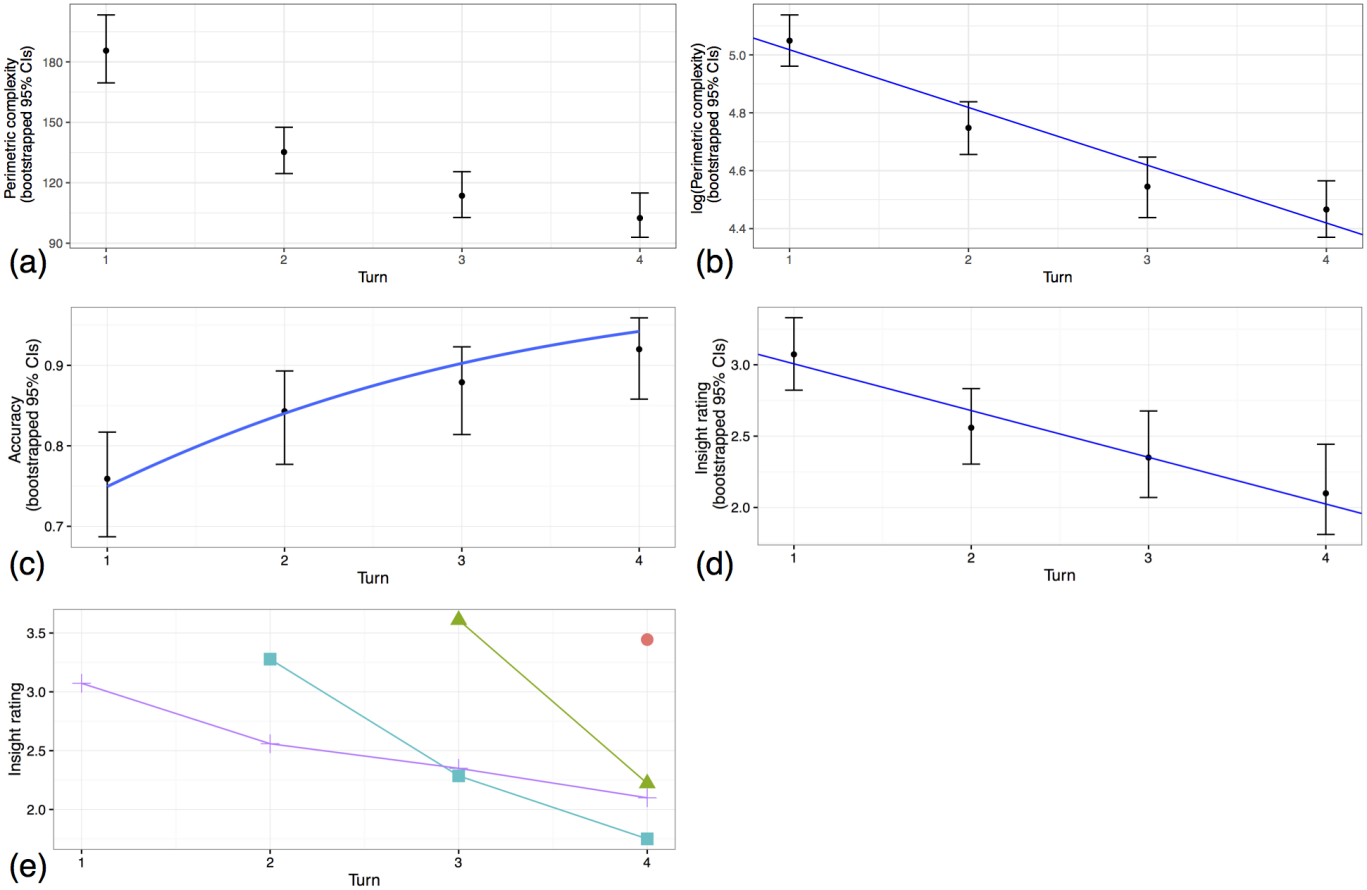
(a) Mean perimetric complexity vs. turn, suggesting a non-linear decrease. (b) Mean log-transformed perimetric complexity vs. turn (dots), with linear mixed-effects model estimate for the effect of turn (line). (c) Mean accuracy vs. turn (dots), with binomial mixed-effects model estimate for effect of turn (curve). (d) Mean insight rating vs. turn for items present since turn 1 (dots), with linear mixed-effects model estimate for the effect of turn (line). (e) Mean insight ratings vs. turn, grouped by the number of the turn in which the items first appeared. When first introduced, the mean insight rating for each group is relatively high. For those groups present for more than one turn (i.e. all items except those introduced during turn 4), there is a subsequent decrease in insight rating.

**Fig 4:**
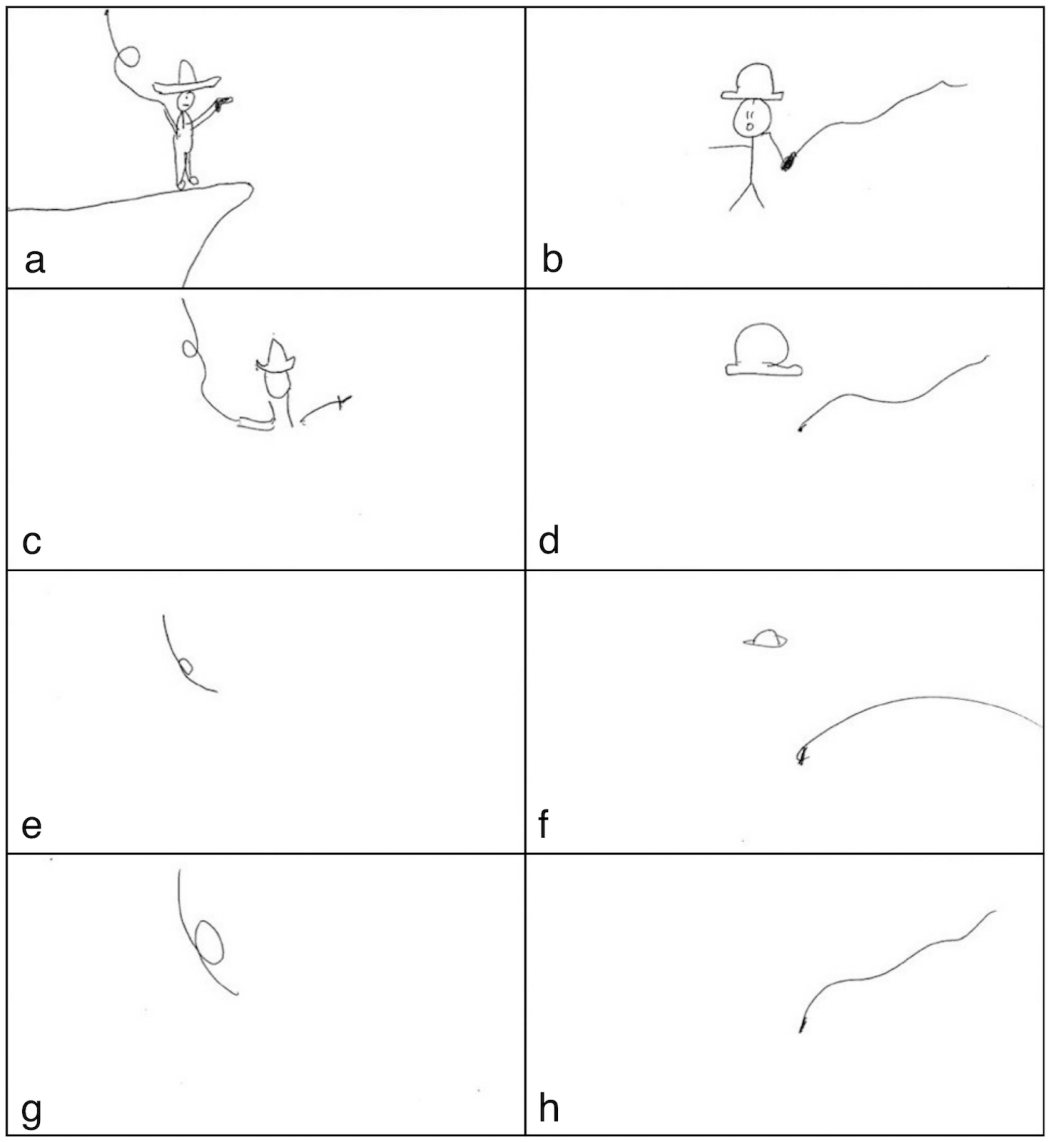
Signals representing cue Harrison Ford across eight rounds for one pair of participants. Rounds increase left-to-right and top-to-bottom, in which case the left column shows drawings by one participant (the signaller in rounds 1, 3, 5, 7) and the right one shows pictures by the other participant (the signaller in rounds 2, 4, 6, 8). Each row thus constitutes one turn.

Garrod et al. [1] measure the visual complexity of a signal by calculating its perimetric complexity [46], which Fay et al. [2] describe as follows: ‘Perimetric complexity is calculated by adding the drawing’s Inside and Outside Perimeter, squaring the result, and dividing by the amount of Ink. Perimetric complexity is a scale-free measure of the graphical information in a picture and is shown to accurately predict the efficiency with which signs of varying complexity are decoded’ (p 376, their footnote 3. A python script for calculating perimetric complexity is available at https://github.com/justinsulik/pythonScripts). They show that symbolisation (as in Fig 1) involves a decrease in perimetric complexity. The data from the present study (Fig 3a) suggests a non-linear decrease, so perimetric complexity is log-transformed for the analyses. Log-transformed perimetric complexity was entered into a linear mixed-effects regression with turn as the fixed effect (random effects structure by-word and by-item: intercepts and slopes for turn). There is a significant negative effect of turn on perimetric complexity (*β* = −0.199, *SE* = 0.033*t* = −6.029, *p* < 0.001, bootstrapped 95% CIs [-0.263, -0.134]). The model shows that perimetric complexity decreases significantly across turns (Fig 3b), meaning that the signals are becoming simpler, as expected (cf. Fig 4).

#### Accuracy

Accuracy (whether the item was guessed correctly or not) was entered as the dependent variable into a binomial mixed-effects regression with turn as fixed effect (random effects structure by-word: intercept only; by-participant: intercept and slope). Accuracy increases significantly across turns, despite the lack of any feedback (*β* = 0.499, *SE* = 0.195, *z* = 2.562, *p* = 0.010, bootstrapped 95% CIs [0.120, 0.933]).

#### Insight

Insight ratings were entered into a linear mixed-effects regression with turn as fixed effect (random effects structure by-word: intercept only; by-participant: intercept and slope). Insight ratings decrease significantly across turns (*β* = −0.328, *SE* = 0.102, *t* = −3.227, *p* = 0.005, bootstrapped 95% CIs [-0.530, -0.130], Fig 3d), meaning that the cognitive mechanisms underlying relevance-deciding inference are recruited more at the start of symbolisation and less so later.

To rule out the possibility that this was merely a fatigue effect, new items were introduced once per turn. If the drop in insight ratings represents a fatigue effect, then ratings for these new items should decrease over turns. If not, insight ratings for the new items should be as high as for items in the first round. Fig 3e illustrates that there is no fatigue effect (indeed, the graph suggests just the opposite—it’s plausible that in later turns, a novel cue was salient compared to cues that had been present for a few turns already).

The absence of a fatigue effect is confirmed by adding a new variable (time) to the above model to represent the number of turns an item has been seen. By the end of turn 3, an item present from the start has been seen in three turns, an item introduced in turn 2 has been seen for two turns, and an item newly introduced in turn 3 has been seen for one turn. If the effect of this new variable overrides the previous effect of turn, then this is no fatigue effect. The model supports this conclusion: the effect of turn drops out (*β* = 0.068, *SE* = 0.067, *t* = 1.020, *p* = 0.308, bootstrapped 95% CIs [-0.068, 0.203]) whereas time is a significant, negative predictor of insight rating (*β* = −0.420, *SE* = 0.115, *t* = −3.637, *p* = 0.001, bootstrapped 95% CIs [-0.652, -0.178]).

To show that this effect is not a proxy for either of the results already observed, insight ratings were entered into a linear mixed-effects model along with accuracy, time and perimetric complexity (log-transformed) as fixed effects (random effects structure by-word: intercept only; by-participant: intercept and slopes for all fixed effects). There is still a significant decrease in insight ratings over time (*β* = −0.362, *SE* = 0.084, *t* = −4.289, *p* < 0.001, bootstrapped 95% CIs [-0.524, -0.194]) when accuracy and perimetric complexity are included as predictors. In this model, accuracy has a significant effect (*β* = 0.367, *SE* = 0.181, *t* = 2.025, *p* = 0.071, bootstrapped 95% CIs [0.009, 0.726]), such that correct guesses were more likely to be accompanied by an ‘Aha!’ experience. This aligns with findings from non-communicative insight tasks [47, 48]. There is no effect of perimetric complexity (*β* = −0.028, *SE* = 0.156, *t* = −0.180, *p* = 0.860, bootstrapped 95% CIs [-0.333, 0.271]), so the decrease in insight ratings is not driven by the visual complexity of the signal.

### Discussion

The data shows a significant decrease in subjective reporting of an ‘Aha!’ experience over time in a graphical novel signalling game. At the very least, this constitutes a predictable behavioural difference along the time-course of symbolisation. Additionally, this behavioural measure is diagnostic of a difference in cognitive processing, and that recruitment of these cognitive mechanisms has previously been observed in novel signalling tasks and in relevance-deciding tasks. In that case, the results show that symbolisation involves a change in the cognitive mechanisms recruited for interpretation, in addition to the previously observed changes in the signal. In particular, earlier stages of symbolisation place more of a burden on the cognitive mechanisms that underlie relevance-deciding inference than later stages.

Given this result, it is not *sufficiently informative* to describe the whole process as relying on induction (this was the most parsimonious hypothesis discussed in the introduction). Firstly, an inductive account makes no predictions regarding the mechanisms of relevance. Secondly, it is by no means clear that induction encompasses the more creative, open-ended problem solving typical of insight. Hypothesis generation (in this case, hypotheses about the meaning of novel signals) is still poorly understood from an inductive point of view [49, 50], but it is also possible that it may be a distinct form of inference entirely [3, 51]. Thirdly, regardless whether insight ever turns out to be amenable to an inductive account, insight problem solving implies distinctive processing mechanisms, and these are demonstrably related to pragmatics [25, 31].

Experiment 1 thus shows a change in cognition during symbolisation, but it remains to be seen what feature of the task predicts this change: parsimoniously, it could be that a decrease in iconicity drives the change in cognition, though the alternative hypothesis considered here is that it is driven by novelty and salience.

## Experiment 2: Task features predicting insight ratings

### Overview

There are several ways to describe the changes during symbolisation. The final rounds of Figs 1 and 4 are less iconic and less complex than the earlier rounds, but they are also more familiar (or conversely, less novel). For instance, the whip in Fig 4 has been present since the start, so in subsequent turns it is increasingly familiar. When it is familiar, people simply recognise it as a problem they’ve already solved. One question, then, is which of these properties (iconicity or familiarity) best predict the extent to which participants rely on relevance-deciding mechanisms or simply recognise the signal.

The following describes how data for iconicity and familiarity were collected, and tests whether either of these predict how insight ratings or accuracy change over time. The prediction is that familiarity will be a better predictor of insight ratings than iconicity.

Although these measures potentially predict the *change* in insight rating over time, another measure (mean Jaccard distance, JD_*μ*_) evaluates how much of a relevance-deciding problem a particular cue item represents *in the first place*. This was based on how predictable the ground is, given the item to be communicated. The prediction is that a higher JD_*μ*_ value (indicating a less code-like, or a more relevance-deciding problem) should lead to an increase in insight ratings the first time an item is presented in the game.

### Methodology

#### Participants

For iconicity and familiarity ratings, participants were recruited via Amazon’s Mechanical Turk (MT) crowd-sourcing platform. Participation was limited to Mechanical Turk workers (called Turkers) who had a higher than 95% approval rating, and who had completed over 1000 tasks on MT. They were paid USD $0.02 per rating. Each Turker was able to provide ratings for one picture or for several pictures as they wished, but the 10 ratings for a signal were always provided by 10 different Turkers. Overall, 295 Turkers provided ratings, and the median number of signals seen by a Turker was 20. Ethics approval was obtained under the University of Edinburgh Linguistics and English Language Ethics committee procedures.

#### Materials

The signals produced during experiment 1 were scanned to yield jpg files (480 × 262 pixels).

#### Procedure: Familiarity and iconicity ratings

For each signal, 10 iconicity ratings and 10 familiarity ratings were collected. The analysis below uses the average of these 10 ratings, so each signal is represented by one iconicity score (an average of 10 Turkers’ iconicity ratings) and one familiarity score (an average of 10 Turkers’ familiarity ratings). The Open Science Framework material includes the images and their scores.

For an iconicity rating, the participants were given the signal and the cue item, and were asked to rate how strongly the signal resembled the cue. They were told that it was drawn during a game of Pictionary, that Pictionary does not require accurate or skillful drawing, and that their ratings should bear this in mind. They selected a rating from a 7-option drop-down list, ranging from ‘extremely bad resemblance’ to ‘extremely good resemblance’.

For a familiarity rating, the participants were given two signals that were produced consecutively (or almost consecutively—see blow) for the same item in the same game, and were asked to rate how strongly the newer signal resembled the older one. The following example refers to the signals from Fig 4. To rate the familiarity of signal *b*, it was presented alongside signal *a*, and participants were asked to rate how similar the two signals were. They selected a rating from a 7-option drop-down list, ranging from ‘extremely different’ to ‘extremely similar’. The higher this resemblance, the more familiar (or less novel) the signal is in the context of the game. Participants were given the same caveats about Pictionary as the iconicity raters.

Two complications were as follows. First, no familiarity rating is possible for signal *a* because this is the first time it appeared in the game, and there is no precedent to compare it to. Thus, familiarity ratings are only possible *after* an item’s first appearance in the game. Secondly, the familiarity of a signal was rated relative to whichever signal from the previous turn it most resembled. Thus, signal *c* would be presented with signal *a* rather than signal *b*. The reason *c* is familiar is that it resembles signal *a*, so its familiarity must be rated relative to *a*. For the same reason, signal *d* would be compared with signal *b*. This procedure produced a rating of how familiar each signal was (apart from the item’s first appearance in the game) based on preceding signals.

#### Procedure: JD_*μ*_ values

Whereas iconicity and familiarity ratings were collected on Mechanical Turk, signals were independently tagged by the author and by a volunteer research assistant to provide JD_*μ*_ measures. Ratings of iconicity or familiarity are very quick, need little instruction and involve at most two images, whereas tagging meaningful elements of the signals is relatively time-consuming; requires detailed coding instructions (and the opportunity to provide feedback on or ask for clarification about those instructions); carefully hypothesising the signaller’s intentions; simultaneously considering multiple images; and reviewing earlier decisions based on later ones. It is thus a great deal more complex and open-ended than rating for resemblance, and less suited to an online platform like Mechanical Turk.

Both taggers followed a detailed coding schema. The main points in the instructions were (1) to tag elements of the signal that the tagger thinks are relevant to guessing the target item (rather than tagging everything recognisable in the signal) and (2) to be consistent, so that the same element appearing in different pictures receives the same tag. For detailed coding instructions and the resulting tags, see Open Science Framework material.

For all possible pairs of new signals drawn for a cue item, the JD (formula 1) was calculated separately for each tagger. These were then averaged to produce a JD_*μ*_ for each item (again, separately for each tagger).

This task is by nature interpretive, and thus allows for substantial variation between taggers. Anyone who has played Pictionary will know that people do not interpret the drawn signals in the same way. The expectation is thus not that both taggers will provide the same tags for a signal, but rather that their resulting JD_*μ*_ measures will correlate strongly. Two features of the coding schema and JD_*μ*_ measure afford a degree of robustness. First, the measure is not content sensitive. It does not matter if one tagger tags the hat in Fig 4 as FEDORA and the other as HAT, as long as they are internally consistent and use the same tag if this element reappears in other signals drawn for this cue. Second, the measure is proportional, so one tagger might provide less detail and another more, since the extra detail will appear both in the numerator and denominator of formula 1.

### Analysis

The main analysis falls into two parts: (1) new items (items on their first exposure, i.e., all items in round 1, and the additional items introduced at the start of each subsequent turn to guard against fatigue effects), and (2) older items (any item after its first exposure). The JD_*μ*_ is only informative for new items, since it reflects how people create a graphical signal based on their world knowledge of the cue, whereas the content of subsequent signals depends in large part on previous signals. On the other hand, the familiarity rating is only possible for older items, since it requires comparison with a previous appearance. Iconicity ratings are available in either case.

### Results

#### New items

There is a high correlation between the two taggers’ by-item JD_*μ*_ measures (*r*_*s*_ = 0.707, *p* < 0.001). The following analysis uses the average of both taggers’ JD_*μ*_ measures, though using either tagger’s measures separately yields the same conclusions.

Averaging by item (since iconicity and perimetric complexity are calculated by signal, whereas the JD_*μ*_ values are calculated by item), there is a moderate negative correlation between JD_*μ*_ and iconicity (*r*_*s*_ = −0.457, *p* = 0.008). Thus, the more iconic an items’ signals, the more consistency there is in how it is represented. There is no relationship between JD_*μ*_ and perimetric complexity (*r*_*s*_ = 0.374, *p* = 0.124) or between iconicity and perimetric complexity (*r*_*s*_ = −0.294, *p* = 0.325).

**Fig 5:**
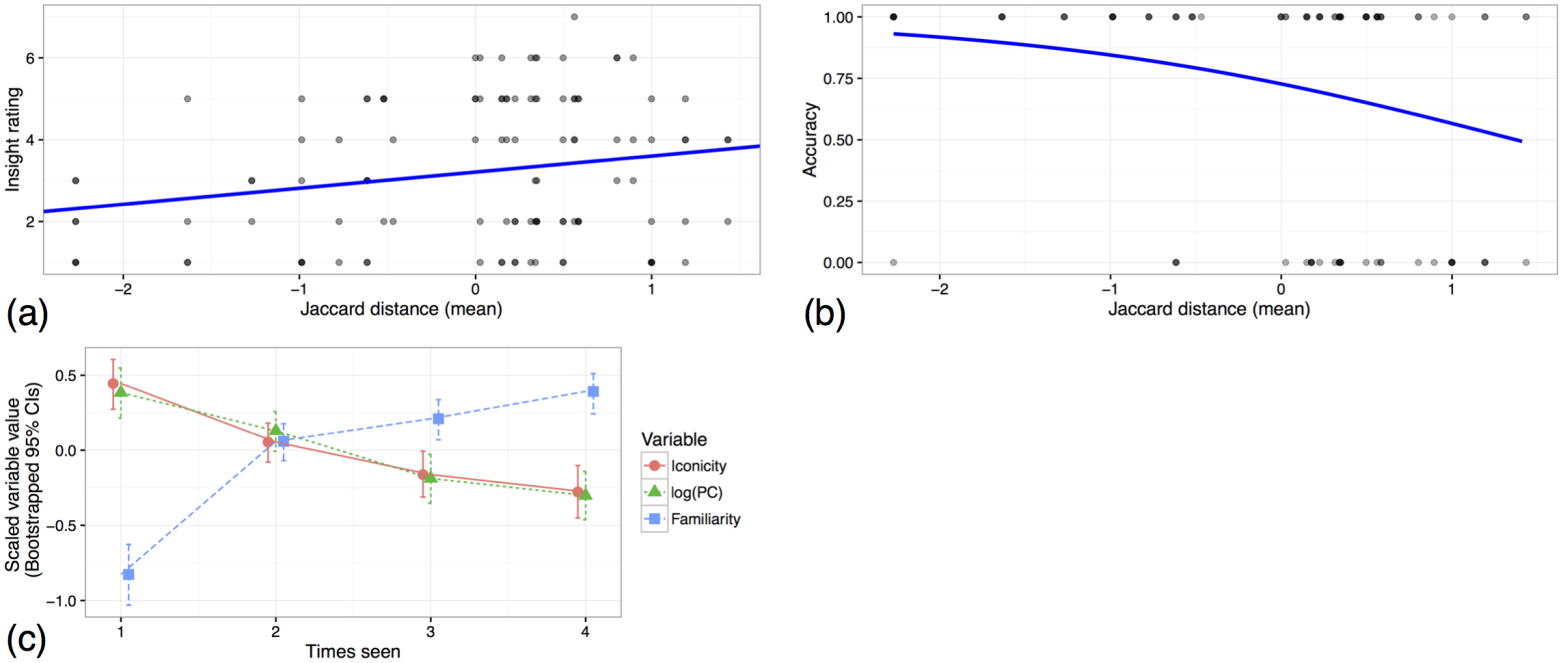
(a) Jaccard Distances (JD_*μ*_ scaled) vs. insight ratings for new signals, with linear mixed-effects model predictions (line). Each dot represents one signal on its first appearance in a game. Since JD_*μ*_ is a by-item measure, but each signal might receive a different insight rating, there are multiple insight ratings per item (and thus per value on the x-axis). (b) JD_*μ*_ (scaled) vs. accuracy for new signals, with binomial mixed-effects model predictions (curve). (c) Mean values for iconicity, perimetric complexity (PC) and familiarity (all scaled) vs. the number of times the item has appeared in a particular game.

Iconicity, parametric complexity and JD_*μ*_ were scaled (after parametric complexity was log-transformed) and entered as fixed effects into a linear mixed-effects model with insight rating as the dependent variable (random effects structure by-participant: intercept and slope for iconicity and parametric complexity. Item word was excluded as a random effect since there is only one JD_*μ*_ measure per word). JD_*μ*_ is a significant, positive predictor of insight ratings (*β* = 0.394, *SE* = 0.165, *t* = 2.389, *p* = 0.019, bootstrapped 95% CIs [0.069, 0.720], Fig 5a). Thus, the less predictable an item’s signals are from its mental representation, the more likely it is that receivers report an ‘Aha!’ moment when inferring the signaller’s intentions. The same conclusion follows using either tagger’s ratings independently, in place of the average just reported (tagger 1: *β* = 0.384, *SE* = 0.173, *t* = 2.215, *p* = 0.029, bootstrapped 95% CIs [0.039, 0.728], tagger 2: *β* = 0.334, *SE* = 0.156, *t* = 2.140, *p* = 0.035, bootstrapped 95% CIs [0.032, 0.649]). However, there was no significant effect of iconicity (*β* = 0.091, *SE* = 0.221, *t* = 0.413, *p* = 0.689, bootstrapped 95% CIs [-0.339, 0.525]) or perimetric complexity (*β* = 0.014, *SE* = 0.171, *t* = 0.080, *p* = 0.940, bootstrapped 95% CIs [-0.331, 0.364]).

Given the relationship between JD_*μ*_ and iconicity, it is worth checking that the lack of an effect of iconicity is not due to their collinearity. However, even when iconicity is the only fixed effect in the model, it still fails to predict insight ratings (*β* = −0.116, *SE* = 0.216, *t* = −0.538, *p* = 0.603, bootstrapped 95% CIs [-0.551, 0.309]). Thus, for novel signals, the recruitment of insight-solving cognitive mechanisms depends on how predictable the ground is given the item, not on perceptual properties such as iconicity or perimetric complexity.

A binomial mixed-effects model with the same fixed effects, but with accuracy as the dependent variable (random effects structure by-participant: intercept only), shows that JD_*μ*_ is a significant, negative predictor of accuracy (*β* = −0.714, *SE* = 0.326, *z* = −2.189, *p* = 0.029, bootstrapped 95% CIs [-1.562, -0.160]) but there is no effect for iconicity (*β* = 0.451, *SE* = 0.279, *z* = 1.619, *p* = 0.105, bootstrapped 95% CIs [-0.080, 1.110]) or perimetric complexity (*β* = 0.145, *SE* = 0.246, *z* = 0.587, *p* = 0.557, bootstrapped 95% CIs [-0.367, 0.696]). Thus, the more of a relevance-deciding problem the item poses, the less likely receivers were to guess it correctly.

As previously, given the collinearity between JD_*μ*_ and iconicity, it is worth checking whether iconicity has an effect on its own. When it is the only fixed effect in the model, it has a significant positive effect (*β* = 0.659, *SE* = 0.246, *z* = 2.673, *p* = 0.008, bootstrapped 95% CIs [0.198, 1.228]), meaning that more iconic signals were easier to guess. Since this effect is overridden by JD_*μ*_ when both are included in a model, performance at novel signalling tasks may sometimes *seem* to reflect an effect of iconicity, but may actually reflect an effect of world knowledge. This suggests that a focus on iconicity in novel signalling tasks may sometimes yield misleading effects.

#### Older items

The focus now shifts to items than have already been seen at least once in a game. JD_*μ*_ is no longer an applicable measure, but familiarity now is. Since some items first appeared in later turns than others, the following uses the time (the number of turns an item has been seen), rather than turn, as a fixed effect.

For older items, there is a moderate correlation between iconicity and perimetric complexity (*r*_*s*_ = 0.367, *p* < 0.001), and a weaker one between iconicity and familiarity (*r*_*s*_ = 0.203, *p* < 0.001). The relationship between perimetric complexity and familiarity is negligible in size (*r*_*s*_ = −0.043, *p* = 0.065).

As a sanity check (to test whether the Mechanical Turk ratings behave as expected), iconicity and familiarity were entered as the dependent variables in two separate linear mixed-effects regressions, with time as the fixed effect (random effects structure by-item: intercepts and slopes for time; by-participant: intercepts only). Since Fig 5c suggests a non-linear effect of time on familiarity, a quadratic term was included. The models show that iconicity decreases significantly over turns (*β* = −0.131, *SE* = 0.019, *t* = −6.952, *p* < 0.001, bootstrapped 95% CIs [-0.168, -0.089]), whereas familiarity increases (*β* = 0.563, *SE* = 0.078, *t* = 7.204, *p* < 0.001, bootstrapped 95% CIs [0.410, 0.712]), though this effect weakens over time given a significant negative quadratic term (*β* = −0.049, *SE* = 0.010, *t* = −5.117, *p* < 0.001, bootstrapped 95% CIs [-0.069, -0.030]). Signals thus become less iconic and more familiar over time, as expected. Although the correlation between iconicity and perimetric complexity is only moderate, their behaviour over time is very similar (Fig 5c).

The main question to be answered here is: which variable best predicts the change in insight ratings? Insight ratings were entered into a linear mixed-effects model as the dependent variable, with iconicity, familiarity and log-transformed perimetric complexity (all scaled) as fixed effects, as well as time (random effects structure by-item: intercept and random slopes for perimetric complexity and familiarity; by-participant: intercept and random slopes for iconicity and familiarity). As observed previously, there is a significant negative effect of time (*β* = −0.087, *SE* = 0.025, *t* = −3.518, *p* < 0.001, bootstrapped 95% CIs [-0.133, -0.037]). In addition, there is a significant negative effect of familiarity(*β* = −0.214, *SE* = 0.082, *t* = −2.624, *p* = 0.016, bootstrapped 95% CIs [-0.372, -0.040]), but no effect of iconicity (*β* = 0.079, *SE* = 0.064, *t* = 1.223, *p* = 0.236, bootstrapped 95% CIs [-0.059, 0.210]), or perimetric complexity (*β* = −0.073, *SE* = 0.053, *t* = −1.378, *p* = 0.169, bootstrapped 95% CIs [-0.176, 0.032]). Thus, the insight ratings decrease as the signals become increasingly familiar (and this does not reduce to an effect of time), but they are not predicted by the signal’s level of iconicity or visual complexity.

To test the effects of the same factors on performance, accuracy was entered as the dependent variable into a binomial mixed-effects regression with the same fixed effects as above (random effects structure by-item and by-participant: intercept only). There is a significant negative effect of perimetric complexity (*β* = −0.761, *SE* = 0.219, *z* = −3.476, *p* < 0.001, bootstrapped 95% CIs [-1.295, -0.315]) and a positive one of familiarity (*β* = 0.462, *SE* = 0.173, *z* = 2.670, *p* = 0.008, bootstrapped 95% CIs [0.072, 0.870]), but no effect of iconicity (*β* = 0.187, *SE* = 0.259, *z* = 0.719, *p* = 0.472, bootstrapped 95% CIs [-0.370, 0.756]) or time (*β* = 0.061, *SE* = 0.096, *z* = 0.632, *p* = 0.527, bootstrapped 95% CIs [-0.144, 0.292]). Thus, guessers are better able to guess the signaller’s intentions as the signals become more familiar and simpler.

## Discussion

To see what factors drive the pattern of responses in experiment 1, measures for familiarity and iconicity were collected, as well the extent to which an item presents a relevance-deciding problem. For new items, higher insight ratings were predicted by a decrease in JD_*μ*_, which measures how predictable the signals were given the item. Over subquent turns, a drop in insight ratings was predicted by an increase in familiarity. Thus, symbolisation can be characterised as a decrease in how much of a burden is placed on the cognitive machinery that underlies human performance on relevance-deciding problems. This shift in processing is not predicted by visual properties of the signal, such as iconicity or visual complexity.

The same factors (familiarity and JD_*μ*_) predict some of the variation in accuracy for guessing new signals, as well as an improvement in accuracy over time. In the former case iconicity is misleading (it has an effect when it is the only regressor, but not once JD_*μ*_ is included), and in the latter case it has no effect. Experimenters should thus account for the extent to which an item presents a relevance-deciding problem in experiment design, or before making claims about the effect of iconicity on performance.

Fig 5a indicates that much of the variation in insight ratings remains unaccounted for, despite the significant effect of JD_*μ*_. It could be that JD_*μ*_ is quite a coarse measure, especially since it is calculated by item rather than by signal. It is, after all, about how the *item* is represented mentally, or the extent to which that representation predicts the contents of drawings that signal the item. However, the question of just how mental representations are translated into *particular* drawings lies outside the scope of the current research. Further, JD_*μ*_ uses a single dimension to quantify the effect of world knowledge, but world knowledge is massively multidimensional. Finally, it could be that there are effects of context (each signal is guessed relative to a particular list of 25 possible items). Despite these limitations, JD_*μ*_ nonetheless has a significant effect in the predicted direction.

A decrease in perimetric complexity predicts an increase in accuracy, in addition to the effect of familiarity. There are several possible interpretations of this (all speculative, alas). Perhaps low perimetric complexity reflects conventionalisation and perhaps accuracy is higher for more conventional items. Perhaps, as signals become similar, they also become more distinct (i.e., less like the signals drawn for other items) and thus easier to guess. These explanations are certainly compatible, but they lie beyond the scope of the present research questions.

One concern about the familiarity ratings is that they involve comparing the signals as a whole (I thank an anonymous reviewer for this suggestion). For instance, in Fig 4, the familiarity rating for signal *b* involves comparing it with signal *a*. There are both similarities and differences between these two pictures. Focusing on the whip might make them seem more similar, whereas focusing on general features (e.g., the layout) might make them seem more different. The worry is that the raters might be doing the latter, and the receiver the former.

The data gathered in this experiment are insufficient to resolve this issue decisively. An eye-tracking study would be interesting to explore the time-course of how receivers narrow in on salient, informative elements of the signal, but that lies beyond the scope of the present research question. However, there are several reasons to think that the above worry does not problematise the conclusions drawn here. First, the raters’ familiarity responses (but not their iconicity responses) predict the receivers’ insight responses. There is thus demonstrably *some* relationship between how the raters are processing the signal for familiarity and how the receiver is inferring its meaning. Second, the receivers’ insight ratings reflect the extent to which they are tackling a relevance deciding problem. It might be that they quickly recognise the whip and make a guess on that basis (i.e., do not need to infer its relevance), or it might be that the whip is not very salient to them, in which case they would be interpreting the picture as a whole, and having to infer the relevance of its parts while inferring the communicative intentions of the signaller. The claim is that the insight rating is a measure of just that. To the extent that the familiarity rating predicts the insight rating, then, it is likely to be unproblematic that familiarity raters are evaluating the signal as a whole.

## General discussion

### Summary

The results show that there is a shift in the cognitive mechanisms recruited for interpretation as a signal changes during symbolisation. This shift is not predicted by visual features of the signal (such as its iconicity or visual complexity), but rather by the extent to which it presents a relevance-deciding problem. Relevance and the inferential mechanisms humans bring to bear on relevance-deciding problems play a role in earlier stages of symbolisation.

The question that prompted this research was whether the change in signals observed during symbolisation implies a concomitant change in cognitive processing, and if so, how to characterise that change. Symbolisation *can* be described as a decrease in iconicity and visual complexity [1]. However, icons can also pose a relevance-deciding problem, in which case symbolisation might *also* involve a decrease in the burden placed on the cognitive mechanisms that humans bring to bear on relevance-deciding problems. In the case of symbolisation, a relevance-deciding problem is characterised by high novelty (or low familiarity), and by a lack of predictability in the signal’s ground. Insight problem solving is the recruitment of a distinctive style of processing to solve hard relevance-deciding problems. Thus, the extent to which something is a relevance-deciding problem should predict the extent to which participants display the hallmarks of insight. Crucially, subjective reporting of an ‘Aha!’ moment is a behavioural hallmark of insight problem solving, and is correlated with the distinctive patterns of insight processing [23, 24]. Subjective reporting of an ‘Aha!’ experience can thus serve as a diagnostic for this mode of processing in a novel signalling task.

The broad hypothesis was that symbolisation involves a concomitant change in cognitive processing, and the specific hypothesis that this is explained by relevance, rather than iconicity or visual complexity. Thus, the prediction was that there should be a decrease in subjective reporting of an ‘Aha!’ experience over time, and that this should be predicted by familiarity, and by how unpredictable an item’s ground is.

To test these hypotheses, participants took part in a repeated novel signalling task. After each guess, receivers provided a rating indicating the extent to which they had experienced an ‘Aha!’ moment. They had practised other insight problems before the experiment, so that they were likely to have a better sense of this experience. Experiment 1 replicated the increase in accuracy and decrease in visual complexity previously demonstrated in the literature on symbolisation [1, 2], showed a decrease in insight ratings over time, and demonstrated that this is not a mere fatigue effect, nor explicable by changes in visual complexity or accuracy. Experiment 2 showed that insight ratings for new items are explained by a measure of how predictable the ground is, given the item (JD_*μ*_), and that the decrease in insight ratings over time is predicted by increasing familiarity, rather than by iconicity or visual complexity. Further, both factors (familiarity and JD_*μ*_) have a significant effect on accuracy, and an apparent effect of iconicity on accuracy was shown to be misleading, since this effect drops out when JD_*μ*_ is included in the model. Thus, both the broad and specific hypotheses were supported by the data.

### Implications

The results have implications beyond this particular experiment. For instance, they align with previously observed differences in the cognitive processing of novel vs. conventional metaphors [26, 52], and with a shift in cognition as novel metaphors become more familiar [27]. Like the novel signals here, novel metaphors present a relevance-deciding problem, and Mashal and colleagues show that there is a strong overlap between the neural mechanisms at work in understanding novel metaphors and those underlying insight problem solving: they both involve activation in right hemisphere temporal lobe areas responsible for searching broad semantic sets, and retrieving less predictable or distantly related semantic information [25]. Similar patterns of activation are found in a novel signalling task [29], but there the patterns of activation are interpreted as indicating the creation or processing of mental models of one’s interlocutor. Thus far, then, the literature has linked novel metaphors and insight [52], or has linked novel signalling and intention reading [29]. The results here suggest that these may all be part of a bigger picture, since they connect insight, novelty, representation, and intention reading. The precise relationship between these various abilities remains unclear for now, but a fuller account of pragmatics and problem solving would require an exploration of how these pieces fit together [25]. Some possibilities are as follows: the cognitive similarity between insight and pragmatics could turn out to be illusory; or the cognitive mechanisms thought to underlie intention reading might be better described as solving relevance-deciding problems; or insight problem solving could co-opt inferential and representational mechanisms initially evolved for pragmatic intention-reading.

Either way, symbolisation implies a particular cognitive trajectory, such that relevance-deciding mechanisms are important for earlier stages of the process. This cognitive trajectory has implications for the evolution of language, since there may have been an iconic protolanguage stage prior to the evolution of symbolic (i.e., conventional) communication in our species [5–9]. Given the various ways iconicity relates to the other variables of interest here, an explanation of how humans evolved language cannot focus solely on properties of the signal (e.g., the extent to which it is iconic), but must also encompass questions of representation and inference. In particular, the results suggest that the cognitive mechanisms of relevance may have been a prerequisite for our ancestors’ novel signalling abilities.

This aligns with the claim that relevance is a fundamental aspect of human communication [12] and that relevance-based pragmatics must have played an explanatory role in the evolution of human language [53]. What the present work adds to such claims is empirical measurement of the extent to which something poses a relevance-deciding problem, and a demonstration that this predicts a difference in cognitive mechanism. However, the results diverge from such claims in terms of how they characterise those mechanisms. Sperber and Wilson [12] argue that interpretation of communicative intentions is based on *deductive* inference, explicitly rejecting the notion that more creative forms of inference underlie interpretation. The results here show that this insistence on deduction is overly restrictive, since insight is a creative form of inference [17, 52] and since insight-like mechanisms play a role in understanding communicative intentions, depending on factors such as novelty and predictability. Similarly, pragmatic inference can be modeled with Bayesian induction [54], but it is by no means clear that induction is able to encompass the creative, open-ended inferences underlying human insight problem solving. The results here thus suggest that two influential models of pragmatic inference (as deductive or inductive) might be incomplete or inaccurate. Regardless, if pragmatics played an important role in early stages of the evolution of language in our species, then the cognitive mechanisms described here played an early role in language evolution.

Language evolution aside, the results have broader implications for iconicity research, since they show that the role of iconicity in processing and performance is sometimes bound up with questions of mental representation. Firstly, iconicity was negatively correlated with JD_*μ*_. Since JD_*μ*_ reflects modes of construal or patterns of salience in world knowledge, it is arguably a measure of complexity in representational structure. Secondly, although it seemed like iconicity drove an increase in accuracy for novel signals, this effect vanished when JD_*μ*_ was included in the model. In this view, what makes Fig 2a a good iconic signal for Harrison Ford is not just that it resembles him, but also that it foregrounds predictably salient aspects of common knowledge about him. The claim about representation, processing and performance coheres with some recent directions in iconicity research. For instance, Emmorey [55] argues that iconicity depends as much on representational structure as it does on perceptual resemblance, and that representational structure must thus play a part in how iconicity affects processing or performance. Ortega and Morgan [56] argue, like Emmorey, that icons are not a homogeneous group, and show that features of representational structure (a sign’s neighbourhood density—the number of associates with distinct meanings) affect the processing of iconic gestures.

Since the novel signals here elicited an ‘Aha!’ response, this offers a new avenue of research for insight problem solving. Historically, research in insight problem solving focused on a small number of hard-to-solve ‘classical’ insight problems (like the marriage problem described in the introduction above). Later, the problem space was expanded with Remote Associates Tasks [57] or Compound Remote Associates problems [17], and even more recently with rebus puzzles [22] and magic tricks [48]. The results here show that novel signalling tasks could be another fruitful way to study insight, at least where they involve relevance. One strong benefit of such a move would be that communication is more central to people’s daily lives than these other problems: they have more experience with trying to communicate in novel ways, it requires no expert knowledge, language is a core human competence, and time to solution is often short.

Finally, there are some ramifications for research into creative aspects of language processing. The results reinforce previous evidence that there is a continuum between relatively conventional and relatively novel meanings, and that variation along this continuum predicts the difference in processing observed here (for instance, in metaphor [26, 27]). Mashal and colleagues describe the continuum in terms of salience, but operationalise salience in terms of familiarity (or conventionality) versus novelty. The current work shows that, in addition to novelty, salience can usefully be operationalised in terms of representational structure. Research in this area could be further enriched by the consideration of such structure. Take the example of ‘crystal river’, a novel metaphor from [52]. It is a relevance-deciding problem since it is novel, so there is no convention to guide interpretation. An interpreter has to infer the relevant feature of a crystal that resembles a river (the ground of the metaphor [58]). Presumably, the ground in this case is that the river is like a crystal in that it is clear or sparkling. It turns out that ‘clear’ is a close associate of ‘crystal’ but that ‘sparkling’ is a more distant associate [59]. If metaphors can vary in terms of how accessible the ground is, it is plausible that this will moderate the effect of novelty on cognitive processing.

### Limitations

**Fig 6:**
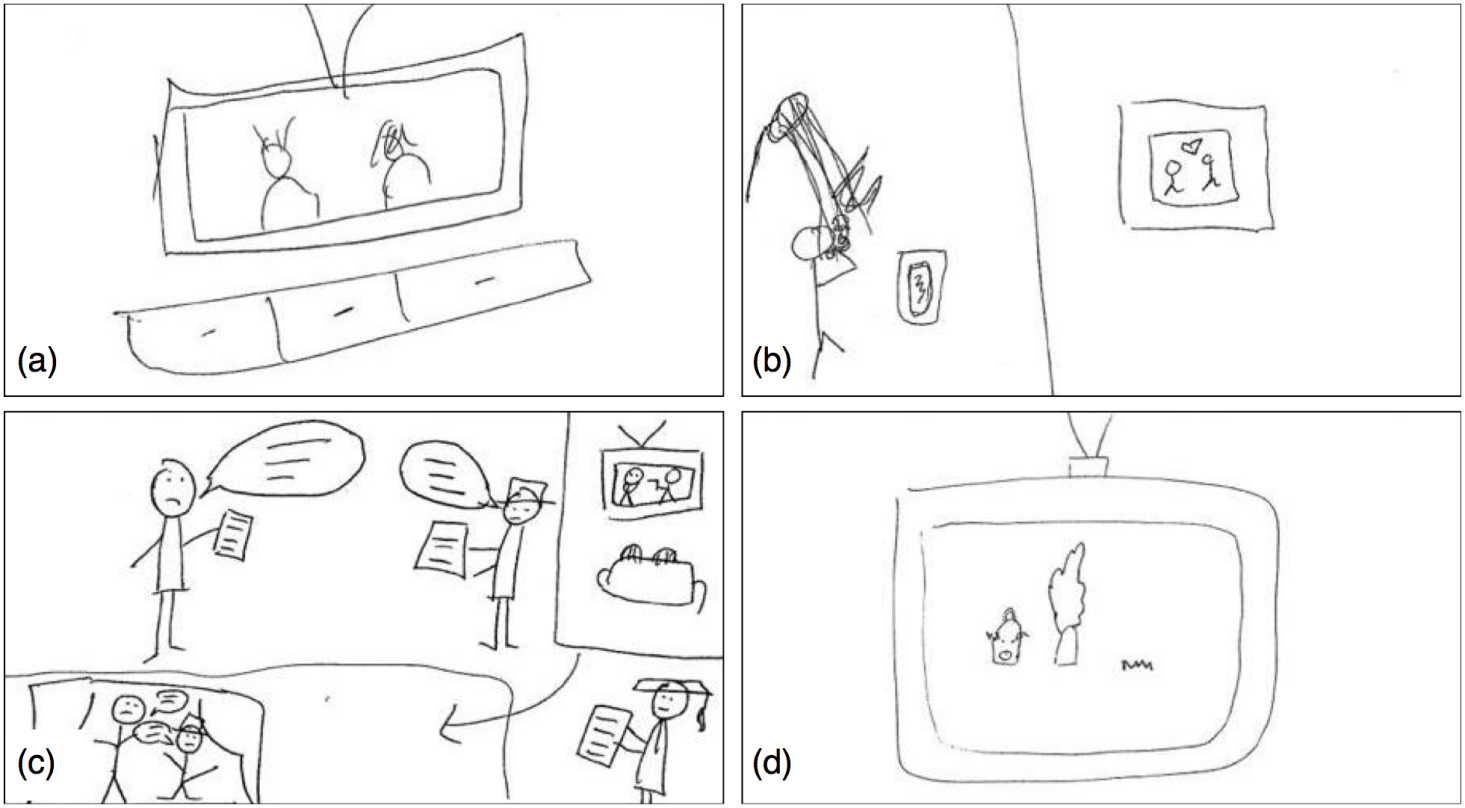
Pictures produced by signallers during experiment 1 representing (a) TV, (b) soap opera, (c) drama, (d) cartoon.

In addition to the above implications for cognitive science, the study also has a couple of limitations. One is that it does not explore contextual effects. Plausible contextual factors are that the set of items is limited to 25 nouns (whereas real-world communication might be more open-ended), and that the context forces an overlap in drawings (‘TV’ appears as an item, but a TV also appears in signals for other items, such as ‘cartoon’ or ‘soap opera’, see Fig 6). A potential worry stemming from these factors is that the measures used here (e.g., ‘Aha!’ ratings and JD_*μ*_ values) might partly reflect contextual constraint (I thank an anonymous reviewer for this suggestion). However, there are several reasons to think that these worries do not undermine the conclusions drawn here.

Sulik [60] explores the effect of contextual constraint on hypothesis generation, and finds that ‘Aha!’ responses increase along with context size (the number of items). In that case, contraining the context to 25 items could mean that participants’ ‘Aha!’ ratings are not as high as they might otherwise have been if this were a more open-ended task. If so, contextual constraint involves a ceiling effect. Thus, higher insight ratings (those at the *start* of the game) might have been even higher in an open-ended task. Since one of the main conclusions here is that the cognitive mechanisms of relevance (as indexed by insight ratings) are important for novel signals, the presence of contextual constraint means that this conclusion is understated, if anything.

Another worry is whether context impacts JD_*μ*_ ratings, and if so, whether this is likely to undermine the conclusions here. Some elements (e.g., TVs and guns) reappear in signals for many items (see Open Science Framework material and Fig 6). In a constrained context such as this, these elements are made less informative by virtue of being shared across items on the 25-item list. If participants aimed to be maximally informative, the contextually constrained task here might actually have fewer of these shared elements than an otherwise similar open-ended task would. In that case, the JD_*μ*_ measure would be affected by context. In contrast to this worry, the claim here is that the signals reflect signallers’ representations of the items, and that JD_*μ*_ is a useful (if noisy) measure of how complex those representations are. In this view, if a TV is central to many people’s representations of an item, then it will probably appear in many signals for that item, and it would not matter to much whether other items also involve TVs.

The issue hinges on how informative signallers manage to be. Sulik and Lupyan [61] show that signallers are typically egocentric when they *generate* novel signals (i.e., they signal based on their own world knowledge, regardless how informative this would be to the receiver). Contextual constraint can drive them to be allocentric (signalling so as to be informative from the receiver’s perspective). However, constraining the *meaning* space (by providing a list of potential target items, as here) has a comparatively small effect on signalling behaviour, whereas constraining the *signal* space (for instance, by forcing signallers to pick from a list of pre-existing signals) has a much larger effect on signalling behaviour. Thus the worry about JD_*μ*_ and context would be better founded if the present study constrained the signal space rather than the meaning space. Further, the small effect seen in [61] involves a very constrained meaning space of five items. The meaning space here is five times larger, so the effect is likely to be even smaller.

The issue of these particular measures aside, the presence of a constrained list need not imply that receivers are going through the list and matching signals with items analytically (i.e., without relevance-deciding mechanisms). Even if the receiver recognises that the signaller has drawn a TV, and even if the constrained context allows them to narrow their guess down to just a few items, they still have to work out whether they are meant to focus on the TV itself, or on what is being shown on the TV, or whether things drawn next to the TV represent things that are shown on TV (Fig 6). These are all relevance-deciding inferences: they involve inferring how to interpret the signal as part and parcel of inferring signaller intention. Overall, then, contextual constraint is unlikely to undermine the conclusions drawn here. Nonetheless, context is an important part of pragmatics, and the precise effect on signalling and interpretation of having a limited set of items, grouped into categories, is worth further exploration.

A final potential issue relating to constraint (though not context in the sense discussed above) is that the taggers for JD_*μ*_ values were told to tag elements they thought were relevant to guessing the item, rather than tagging all elements of the signal. However, this plausibly affects the JD_*μ*_ values (I thank an anonymous reviewer for raising this question).

On the practical side, this instruction to taggers was intended to minimise subjectivity and noise, for several reasons. Firstly, the tags are meant as a measure of the signalers’ mental representations of the item, so it is reasonable that taggers be aware of the item. The point of the tags is, for instance, to measure the extent to which a whip is a highly predictable element of various signals for Harrison Ford (and it turns out to be quite predictable). Secondly, as anyone who has played Pictionary will know, the guesses produced can be wide and varied. If taggers did not know what the image was meant to represent, there would be a great deal more noise in the tags. Thirdly, without this constraint, the tags would suffer greatly from the *Gavagai* problem [62]: there might be multiple ways to construe each element, but the JD_*μ*_ value is meant to reflect the *signalers’* construals.

Practical considerations aside, there are data-supported reasons for this choice. Sulik and Lupyan [61] show that people vary quite widely in how informative they are when generating signals, but are a lot more consistent when evaluating signals. Thus, by having the taggers evaluate what they thought were relevant elements, given the item, they are thus demonstrably more likely to be consistent. Nonetheless, there is a fair amount of variation in the tags, though the observed relationship between JD_*μ*_ and ‘Aha!’ ratings holds regardless which tagger’s tags were used to derive a JD_*μ*_ measure for each item.

There are other limitations that do not relate to constraint or context. Just how humans understand conventional symbols is an important question, but the experiments here do not seek to explore or characterise those cognitive processes. Instead, the focus here has been on finding something useful to say about what happens, cognitively, when signals are *not* conventional or familiar. One strong candidate for the non-insight cases is Bayesian induction [63]. Though Fay et al. [2] do not explicitly address the issue, they mention that inductive biases play a role in symbolisation. The results here can be interpreted to mean that, even though induction may play a very large role, it cannot explain *all* stages of symbolisation. Similarly, the experiments here do not speak to the question of how people recognise things on sight (for those images that obviously resemble their referents). That is a very broad question indeed, but in as far as any distinction can be made between perception and inference (and it might be that no clear-cut distinction can be drawn), this seems to be a matter of perception.

Second, the experiments consider just one type of novel signalling task: graphical signals. Graphical signals differ from other novel signals (such as spontaneous gesture) in several ways. For instance, they are permanent (the receiver can review the entire picture in any order at any time, whereas a novel gesture is more temporary). Relatedly, they are more transparent (it is easier to see what kind of hat is being drawn in Fig 2a than it would be if gestured, where shape of the whole hat cannot be perceived simultaneously).

JD_*μ*_ is not a general measure of representational structure. It only measures the amount of variation in graphical signals produced independently for the same cue (in order to see how predictable those signals are given the cue). Different values might be obtained by applying the same strategy to gestural signals, or by applying a different strategy (such as by collecting word association data).

The familiarity rating just measures how familiar a signal is given the previous signal. It thus does not reflect cumulative familiarity with a signal over several rounds. Nor does it measure increasing familiarity with the *items* themselves as the game progresses. None of these limitations problematise the conclusions drawn here, though.

It might be objected that subjective reporting of an ‘Aha!’ experience could be merely a surprisal effect (i.e., an indication that participants are surprised to see something new, rather than indicating the recruitment of particular cognitive mechanisms for relevance-deciding inference). This is unlikely to be problematic for several reasons. Firstly, surprisal and ‘Aha!’ ratings are at best weakly related (*r*_*p*_ = 0.06, in [48]). Secondly, not only does subjective reporting of an ‘Aha!’ experience correlate with activation in the cognitive networks associated with insight, but this activation has also been shown to be depend on variables tested here, such as novelty or predictability. Thirdly, insight shows distinctive patterns of neural activity *prior* to solution, involving resting brain state [64], or the suppression of irrelevant information [65, 66]. Since these differences are observable prior to solution, they cannot be explained by the surprise that arises when the solution becomes conscious. Finally, behavioural evidence shows that insight problem solving is characterised by distinctive patterns of attention [67–70]; executive control [71]; error (e.g., errors of commission vs. omission [64]); or memory [72]. Even physiological measures such as heartbeat distinguish insight and from non-insight problem solving [73].

Recent work suggests that the distinction between insight and analytic processes is not always clear cut [74, 75] or that analytic and insight processes may complementarily tackle different aspects of a problem [75]. The claim here is just that communicative problems can place more or less of a burden on relevance-deciding mechanisms, that insight problem solving lies at one end of this spectrum, and that we are more likely to observe hallmarks of insight problem solving (such as the ‘Aha!’ experience) when more of a burden is placed on those mechanisms. This claim thus does not rely on a clear-cut distinction between insight and non-insight processes.

## Conclusions

Symbolisation involves a shift from complex, iconic signals to simpler, less iconic signals, but it also involves a shift in cognitive processing that is not predicted by iconicity or visual simplicity. In early stages of the process, more of a burden is placed on the cognitive mechanisms that humans bring to bear on relevance-deciding problems, but this burden decreases over the time. These mechanisms underly creative insight problem solving, but they are also active when a signal poses a relevance-deciding problem, such as novel metaphor or novel signals here.
